# Improving Appropriate Use of Medical Masks for COVID-19 Prevention: The Role of Face Mask Containers

**DOI:** 10.4269/ajtmh.20-0886

**Published:** 2020-08-04

**Authors:** Dimie Ogoina

**Affiliations:** Infectious Disease Unit, Department of Internal Medicine, Niger Dela University/Niger Delta University Teaching Hospital, Yenagoa, Bayelsa, Nigeria

## Abstract

Use of medical masks is a key strategy for COVID-19 prevention among healthcare workers. Unfortunately, there are global shortages of this essential commodity, and many have resulted in inappropriate usage to conserve supply. This article highlights the likely benefits of face mask containers in promoting safe, appropriate, and extended use of medical masks by healthcare workers in settings where a sustainable supply of medical masks may be limited.

Public wearing of face masks is now one of the key strategies for containing the current COVID-19 pandemic across the globe.^1,2^ The WHO has also recommended targeted continuous use of medical masks by healthcare workers working in clinical areas in health facilities in geographical areas with community transmission of COVID-19.^2^ According to the WHO, face masks are used appropriately when they always cover the mouth and nose, when the front and inside of the face mask is not touched, and when hand hygiene is performed before wearing, and after touching and removal of the face mask.^2^ During targeted continuous mask use, healthcare workers are required to wear medical masks throughout their entire shift and advised to replace their medical masks when wet, damp, visibly soiled, damaged, and if the health worker/caregiver removes the mask (e.g., for eating or drinking or caring for a patient who requires droplet/contact precautions for other reasons).^2^

Face mask use has been shown to be associated with a large reduction in the risk of COVID-19 infection.^3^ Unfortunately, it is not comfortable to wear face masks for prolonged periods.^2,4^ As an infectious disease physician working in a COVID-19 isolation facility in Nigeria, I have observed that most healthcare workers do not have adequate supplies of medical masks to replace them each time there is need to temporarily remove their masks to undertake activities such as eating or drinking or when alone in their offices or cars. Many have therefore opted to wear masks on their chin and neck or keep potentially contaminated face masks on desks, or inside pockets, or bags in close contact with other personal belongings.^5^ In most cases, the masks are handled carelessly and squeezed repeatedly during removal and reuse. The consequence of this inappropriate use of face masks is self- and environmental contamination and increased risk of transmission of COVID-19.

Although there is a growing literature on strategies to decontaminate and reuse single-use medical masks,^4,6^ the WHO currently does not recommend reuse of single-use medical masks. However, if medical masks are to be worn continuously by healthcare workers for up to 8 hours or more every working day, then there should be provision to temporarily and safely store them for extended use during the day, especially when they are not visibly soiled, wet, damp, or damaged.

The use of face mask containers could help to promote appropriate and safe storage of face masks and facilitate extended use when medical masks are not due for replacement. We designed a plastic box and a leather pouch as face mask containers (Figure 1) to store masks temporarily when not in use. Within the containers, the masks are secured by their straps and held flat in their natural positions without the risk of squeezing and self- and environmental contamination. This way they can easily be picked up by the straps and worn safely without touching the front or inside of the mask. We also created vents on both sides of the face mask containers to improve ventilation in the containers when closed.

**Figure 1.**
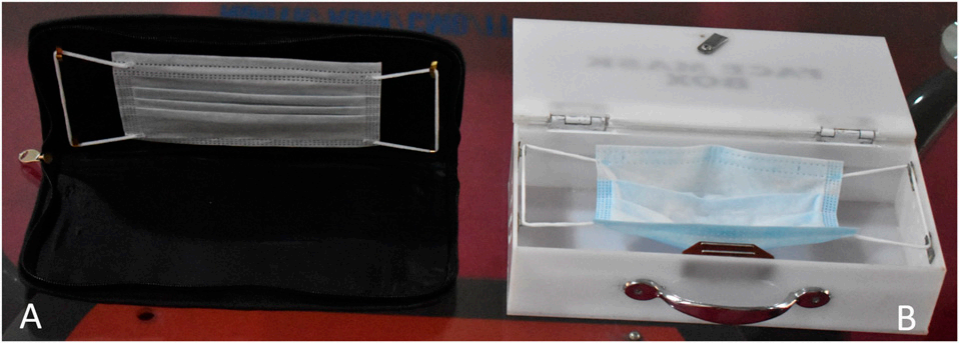
Face mask containers containing surgical masks. Leather pouch (**A**) and plastic box (**B**) designed as face mask containers to safely store face masks to avoid self- and environmental contamination and promote extended use of face masks in the face of scarcity.

In the wake of global supply shortages,^7^ appropriately designed face mask containers could be useful in promoting safe extended use of medical masks, especially in resource-limited healthcare settings. Face mask containers could conserve limited supplies of medical masks for sustainable use by frontline healthcare professionals. Compared with the chin, neck, pocket, or desk, a face mask container is probably a safer environment to store a medical mask when there is need to remove it temporarily to perform activities such as eating or drinking, or when there is need to temporarily expose the mouth and nose to relieve the discomfort associated with the prolonged use of face masks.

Face mask containers should however not be used to store medical masks that are due for replacement, especially when they are wet, damp, visibly soiled, or damaged. Unfortunately, an unintended risk of these containers is the likelihood of prolonged storage and repeated reuse of masks that should have been discarded or washed before reuse.

With the growing call for universal masking as a key cost-effective strategy to combat the COVID-19 pandemic, it is my view that the benefits of face mask containers in promoting appropriate use of masks and enabling extended and safe use far outweigh the risks. Further studies on the benefits and risks of face mask containers would be useful to confirm these assertions.
